# Facile Approach to Prepare rGO@Fe_3_O_4_ Microspheres for the Magnetically Targeted and NIR-responsive Chemo-photothermal Combination Therapy

**DOI:** 10.1186/s11671-020-03320-1

**Published:** 2020-04-17

**Authors:** Chunyong Liang, Jiying Song, Yongguang Zhang, Yaping Guo, Meigui Deng, Wei Gao, Jimin Zhang

**Affiliations:** 1 Research Institute for Energy Equipment Materials, Tianjin Key Laboratory of Materials Laminating Fabrication and Interface Control Technology, School of Materials Science & Engineering, Hebei University of Technology, Tianjin, 300130 China; 2 Hebei Key Laboratory of Functional Polymers, National-Local Joint Engineering Laboratory for Energy Conservation of Chemical Process Integration and Resources Utilization, School of Chemical Engineering and Technology, Hebei University of Technology, Tianjin, 300130 China; 3 Key Laboratory of Cancer Prevention and Therapy, Department of Interventional Therapy, Tianjin Medical University Cancer Institute and Hospital, National Clinical Research Center for Cancer, Tianjin’s Clinical Research Center for Cancer, Tianjin, 300060 China

**Keywords:** rGO@Fe3O4 microspheres, NIR response, Magnetic target, Chemo-photothermal therapy

## Abstract

Near-infrared (NIR)-light responsive graphene have been shown exciting effect on cancer photothermal ablation therapy. Herein, we report on the preparation of Fe_3_O_4_-decorated hollow graphene microspheres (rGO@Fe_3_O_4_) by a facile spray drying and coprecipitation method for the magnetically targeted and NIR-responsive chemo-photothermal combination therapy. The microspheres displayed very high specific surface area (~ 120.7 m^2^ g^−1^) and large pore volume (~ 1.012 cm^3^ g^−1^), demonstrating distinct advantages for a high loading capacity of DOX (~ 18.43%). NIR triggered photothermal effect of the rGO@Fe_3_O_4_ microspheres responded in an on-off manner and induced a high photothermal conversion efficiency. Moreover, The Fe_3_O_4_ on the microspheres exhibited an excellent tumor cells targeting ability. The chemo-photothermal treatment based on rGO@Fe_3_O_4_/DOX showed superior cytotoxicity towards Hela cells in vitro. Our studies indicated that rGO@Fe_3_O_4_/DOX microcapsules have great potential in combined chemo-photothermal cancer treatment.

## Introduction

Cancer is one of the most malignant diseases in the world and is a leading cause of human death [1, 2]. Although chemotherapy is commonly used in the clinic cancer treatment, several key issues including low therapeutic efficiency and extensive side effects seriously limit its application [3]. Drug delivery systems (DDS) have shown great advantages in enhancing drug solubility, bioavailability, and tumor accumulation, which are expected to prominently improve their antitumor efficiency [4]. Recently, hollow microspheres employing as drug delivery systems have gained increasing attention owing to their large surface area and abundant porous structures [5–8], and several hollow microsphere materials have been designed with innovative technologies [9–13].

Graphene oxide (GO), a new type of inorganic free-metallic material, has been widely investigated in drug delivery due to its unique features, such as good biocompatibility, low cost, and simple preparation [14–17]. Notably, graphene oxide can effectively transform light to heat when triggered by NIR irradiation [18–20], becoming a promising strategy to improve the photothermal therapy effect of cancer. Chen group has reported that GO could deliver the anticancer drugs by non-covalent interaction such as π-π stacking, hydrogen bonding, and electrostatic adsorption [21]. However, the 2D graphene oxide nanosheet tends to agglomerate due to the large specific surface as well as the van der Waals bonds among the graphene layers [17, 22], resulting in poor solubility in water and decreasing drug loading ability. Some strategies have been explored to overcome these shortcomings. Tsukruk group has developed a graphene hollow capsules using layer-by-layer assembly technology [23], which showed an extremely high drug loading compared to other GO materials. This could be contributed to the high specific surface area and large pore volume of the hollow capsule stabilized by GO. However, few reports have referred to the study of GO with a three-dimensional-connected pore structure for drug delivery.

Although many reported drug delivery systems have exhibited superior drug loading ability and controlled drug release behavior, their preclinical research and applications are also limited due to an insufficient specificity to target tumor tissues [24]. Among various drug target delivery systems, Fe_3_O_4_, a magnetic-target material is wildly used in cancer therapy for its high magnetic responses, stable quality, and easy achievement [25–29]. Ni group has developed an Fe_3_O_4_@SiO_2_ core-shell structure nanoparticles with superparamagnetic property for magnetic targeting of tumors [30]. Furthermore, Fe_3_O_4_ anchored GO nanoparticles have been well studied in combination of magnetic target delivery and photothermal therapy [31–34].

**Scheme 1:**
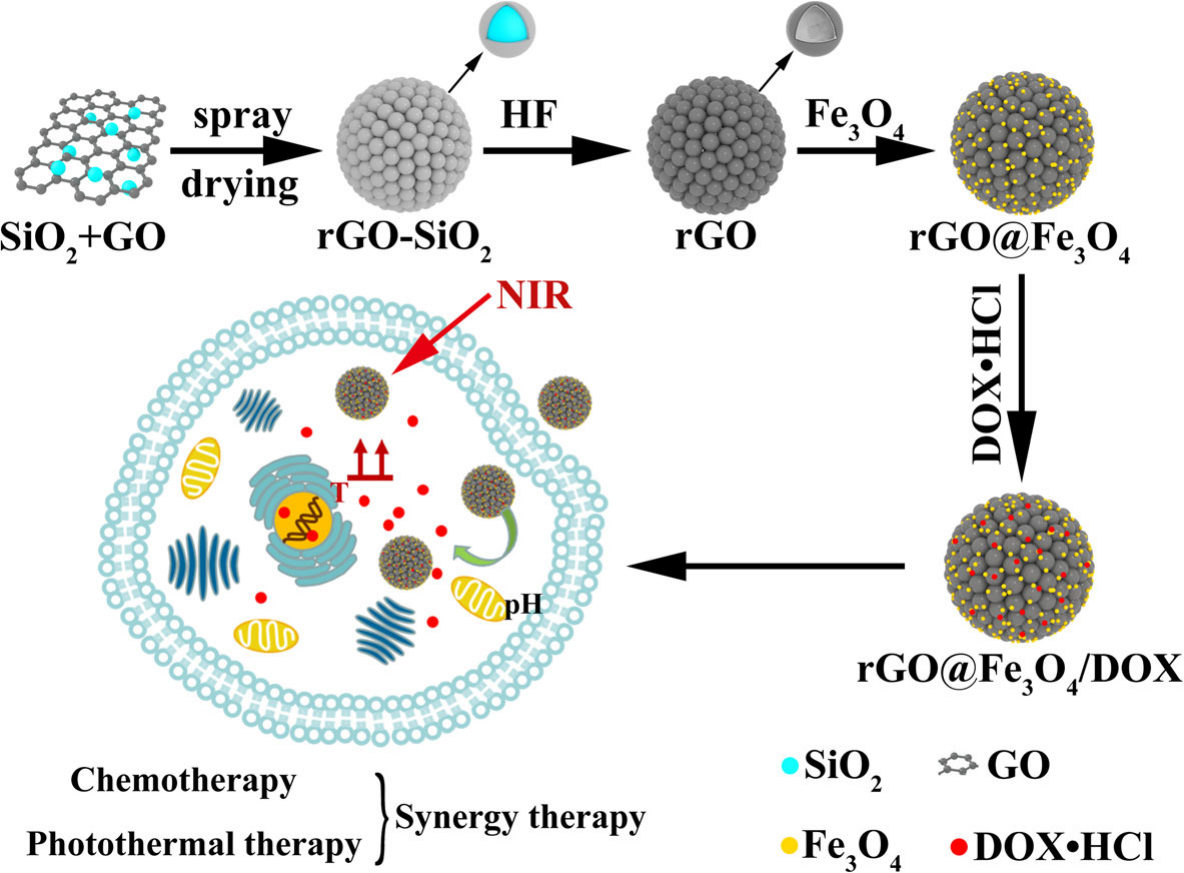
Schematic illustration of rGO@Fe_3_O_4_/DOX microspheres and the combined photothermal-chemotherapy for tumor inhibition

In the present study, we report an advanced strategy for developing a DDS platform comprising an iron oxide decorated rGO hollow microspheres (rGO@Fe_3_O_4_) for the magnetically targeted and NIR triggered photothermal therapy (PTT). As depicted in Scheme 1, rGO@Fe_3_O_4_ hollow microspheres were prepared through three steps. Firstly, rGO-SiO_2_ is synthesized by spray drying method using SiO_2_ as a template and then rGO hollow microspheres were obtained by removing SiO_2_ with HF etching. Afterward, Fe_3_O_4_ nanoparticles were anchored onto rGO hollow microspheres to construct rGO@Fe_3_O_4_ microspheres. In this system, rGO is served as a NIR-triggered PTT agent, and Fe_3_O_4_ can offer the magnetic targeting property towards to Hela cell. Doxorubicin (DOX), encapsulated microspheres (rGO@Fe_3_O_4_/DOX) based on pore adsorption and π-π stacking, is expected to exhibit ultrahigh drug loading capacity and pH-responsive drug release behavior, and can significantly enhance the anticancer effect for the combination of photothermal-chemotherapy.

## Materials and Methods

### Materials

Iron chloride hexahydrate (FeCl_3_·H_2_O), sodium hydroxide (NaOH), and ferrous sulfate heptahydrate (FeSO_4_·7H_2_O) were purchased from Sinopharm Chemical Reagent Co., Ltd. Hela cells are from Tianjin Cancer Hospital. Phosphate buffered saline (PBS)_,_ Doxorubicin hydrochloride (DOX·HCl), Dulbecco’s minimum essential medium (DMEM), 4',6-diamidino-2-phenylindole (DAPI), and cell counting kit-8 (CCK-8) were purchased from Solarbio Science and Technology Co., Ltd. SiO_2_ (~ 300 nm) was purchased from Shanghai Yuanjiang Chemical Company. Graphene oxide deion water solution (2 mg/ml) was a commercially available product from Nanjing Xianfeng Company.

### Preparations of rGO@Fe_3_O_4_ Microspheres

Hollow graphene microspheres were prepared via spray drying method using SiO_2_ (300 nm) as the template. Briefly, 100 mL SiO_2_ suspension liquid (50 mg mL^−1^) was slowly dropped into 300 mL GO aqueous solution (2 mg mL^−1^) under drastic stirring, the mixed solution was spray dried at 200 °C in a spray dryer unit. Subsequently, the product was kept at 300 °C under Ar protection for 2 h and rGO-SiO_2_ was obtained. To remove SiO_2_, rGO-SiO_2_ was placed in HF solution (10%) for 48 h at 60 °C. The solid product was washed several times and dried in a vacuum drying oven at 60 °C for 12 h, rGO was finally obtained with a yield of 75%.

The rGO@Fe_3_O_4_ nanoparticles were prepared via the coprecipitation method. In a typical process for the synthesis of rGO@Fe_3_O_4_ nanoparticles, 0.27 g FeCl_3_·H_2_O, 0.28 g FeSO_4_·7H_2_O, and 0.1 g rGO hollow microspheres were dissolved in 10 mL deionized water and stirred for 30 min at 50 °C. Then, 60 mL NaOH (0.15 mol L^−1^) was slowly added under continuous stirring at 50 °C for 12 h. The products were finally separated magnetically and washed repeatedly with deionized water and ethanol several times followed by drying at 60 °C under vacuum for 12 h.

### Structural Characterization

The size and morphology of the sample were analyzed using a field emission scanning electron microscopy (FE-SEM, Hitachi, S-4800) and transmission electron microscopy (TEM, JEM2100F, JEOL). The composition of the products was analyzed via X-ray diffraction system (XRD, D8 Focus, Cu Ka radiation, Bruker, Germany) at a scan rate of 12 °/min range from 10 to 80°. Also, X-ray photoelectron spectroscopy (XPS) was carried out on a XPS spectrometer (Thermo Fisher Scientific, ESCALAB 250Xi, America). The FTIR (FT-IR, AVATAR360, Nicolet) were recorded from 500 to 4000 cm^−1^ at a resolution of 4 cm^−1^. Magnetic measurements were performed using a superconducting quantum interference device (SQUID, Quantum Design MPMS) magnetometer at room temperature (300 K). The Raman spectra were collected using a Raman spectroscope (Renishaw, inVia Reflex, England) with a 532 nm wavelength laser. The content of rGO was evaluated using a thermogravimetric analyzer (TGA, TA Instruments-water LLC, SDTQ-600). The specific surface area was measured using the Brunauer-Emmett-Teller (BET) technique. UV-Vis spectra were recorded using a Beckman DU 800 nucleic acid/protein analyzer (Beck-man Instruments, Inc., Rosemead, CA).

### DOX Loading and Release

DOX, a model chemotherapeutic drug doxorubicin, was encapsulated into the cores of rGO@Fe_3_O_4_ to evaluate the loading and releasing behaviors of anticancer drugs in vitro. rGO@Fe_3_O_4_/DOX was prepared according to the previous reference. In brief, 10 mL (0.2 mg mL^−1^) of DOX aqueous solution was added to 10 mg of rGO@Fe_3_O_4_ solution, the mixture was ultrasonically homogenized to insure no significant precipitation. Then, the mixture was equilibrated on a reciprocating shaker (SK-O180-Pro) at a speed of 150 rpm for 24 h. After centrifugation at 6000 rpm for 10 min, unloaded DOX was removed, the supernatant of rGO@Fe_3_O_4_/DOX was measured via UV-Vis spectrophotometer to determine the amount of DOX loaded. The OD of DOX was recorded at 490 nm, the following equations were used to calculate the loading efficiency (LE) and loading capacity (LC) of the DOX:

$$ \mathrm{LE}=\left(\mathrm{total}\ \mathrm{amount}\ \mathrm{of}\ \mathrm{DOX}-\mathrm{Free}\ \mathrm{DOX}\right)/\mathrm{total}\ \mathrm{amount}\ \mathrm{of}\ \mathrm{DOX} $$

$$ \mathrm{LC}=\left(\mathrm{total}\ \mathrm{amount}\ \mathrm{of}\ \mathrm{DOX}-\mathrm{Free}\ \mathrm{DOX}\right)/\mathrm{amount}\ \mathrm{of}\mathrm{rGO}@{\mathrm{Fe}}_3{\mathrm{O}}_4/\mathrm{DOX} $$

The in vitro release studies of DOX was performed by putting rGO@Fe_3_O_4_/DOX (10 mg) in a dialysis bag (MWCO = 1000) with phosphate-buffered saline (PBS, 30 mL) at pH 5.4, 6.5, or 7.4, placing it in a 37 °C water bath and shaking at 80 rpm. At predetermined intervals, 3 mL of the release medium was collected and the amount of released DOX was calculated by measuring the UV-Vis at 480 nm.

### NIR-Triggered Photothermal Effect of rGO@Fe_3_O_4_ Microspheres

To monitor the influence of rGO@Fe_3_O_4_ dose on NIR-triggered photothermal effect, the rGO@Fe_3_O_4_ solutions with different concentrations (0.0625, 0.125, 0.25, 0.5, and 1 mg mL^−1^) were irradiated with NIR laser at 2 W cm^−2^ for 5 min, respectively. Furthermore, the influence of NIR energy on the photothermal effect was evaluated by irradiating rGO@Fe_3_O_4_ (0.25 mg mL^−1^) with different powers (1 W cm^−2^, 1.5 W cm^−2^, 2 W cm^−2^) for 5 min. The real-time temperature was measured using a FLIR I5 infrared thermal camera.

### In Vitro Uptake

Hela cells were seeded in 35 mm^2^ confocal dishes at a density of 1 × 10^5^ cells/well. After incubating for 24 h in an incubator (5% CO_2_, 37 °C), the medium was removed, and the fresh medium containing rGO@Fe_3_O_4_/DOX microspheres and rGO@Fe_3_O_4_/DOX with magnet were added and cultivated for another 5 h. The rGO@Fe_3_O_4_/DOX concentration was 0.1 mg mL^−1^. The cells were then washed three times with cold PBS (pH = 7.4) and fixed with 4% paraformaldehyde solution for 20 min (CLSM, TCSSP5II, Leica, Ernst-Leitz-Strasse, Germany).

### Cell Viability Assays

The cytotoxicity of these microspheres was evaluated by a CCK-8 assay after NIR treatment. HeLa cells were seeded onto 96-well plates (5 × 10^3^ cells/well) in 100 μL of the medium and cultured in 5% CO_2_ at 37 °C for 24 h. For the biocompatibility assessment, rGO@Fe_3_O_4_ were added to the well with a concentration range from 0.01 to 0.2 mg mL^−1^; for the single photothermal therapy group, rGO@Fe_3_O_4_ was added with a concentration range from 0.01 to 0.2 mg mL^−1^, and applying NIR light irradiation for 10 min (2 W cm^−2^, 808 nm); for the combined photothermal-chemotherapy group, rGO@Fe_3_O_4_/DOX was added with a concentration range of rGO@Fe_3_O_4_/DOX from 0.01 to 0.2 mg mL^−1^, and applying NIR light illumination for 10 min (2 W cm^−2^,808 nm). The cells were proceeding incubated for 24 h or 48 h. Afterward, the cells were washed with PBS and incubated in 100 μL DMEM medium containing 10 μL CCK-8 solution for another 40 min. The viability was detected using a microplate reader at a wavelength of 450 nm. All the experiments were conducted in triplicate.

## Results and Discussions

### Synthesis and Morphology Characterization

**Fig. 1:**
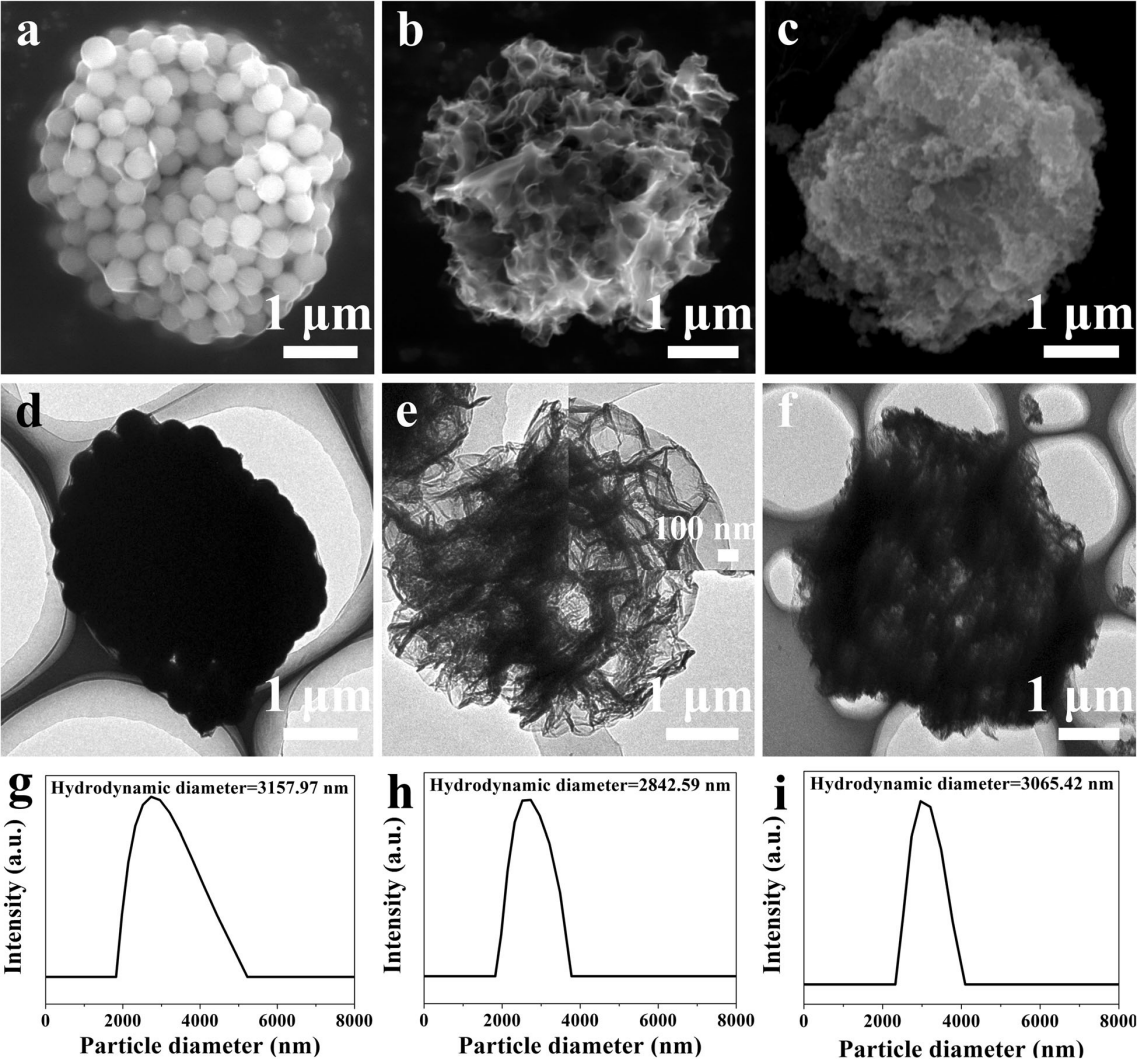
Morphology characterization of microspheres. SEM images of (**a**) rGO-SiO_2_, (**b**) rGO, (**c**) rGO@Fe_3_O_4_; TEM images of (**d**) rGO-SiO_2_, (**e**) rGO, (**f**) rGO@Fe_3_O_4_; hydrodynamic size distribution of the corresponding samples (**g**) rGO-SiO_2_, (**h**) rGO, (**i**) rGO@Fe_3_O_4_

The preparation of rGO@Fe_3_O_4_ microspheres was performed through three steps. Firstly, rGO-SiO_2_ microspheres were synthesized by spray drying using SiO_2_ as a template. The morphology of rGO-SiO_2_ microspheres was characterized by SEM and TEM. As shown in Fig. 1a, the rGO-SiO_2_ microspheres with diameters of 3 μm exhibited uniform spherical shape and comprised of many crowded SiO_2_ nanoparticles (~ 300 nm). The TEM data and the hydrodynamic diameter measured by dynamic light scattering also confirmed the results. (Fig. 1d, g). Then, hollow rGO microspheres were obtained by removing SiO_2_ from rGO-SiO_2_ with heating at 300 °C and HF etching. Obvious pores with a pore size of about 300 nm could be observed due to SiO_2_ dissolution (Fig. 1b, e). Finally, Fe_3_O_4_ in virtue of magnetic targeted ability was decorated onto the porous rGO by the coprecipitation method. The observation of SEM and TEM illustrated that the remarkable decrease of pore size after Fe_3_O_4_ loading was obtained, (Fig. 1c, f), providing the feasibility of drug delivery and the controlled drug release. Notably, the particle size and hydrodynamic size distribution of rGO-SiO_2_, rGO, rGO@Fe_3_O_4_ have no more visible changes during these treatments (Fig. 1g, h, i).

### Structure and Composition Characterization

**Fig. 2:**
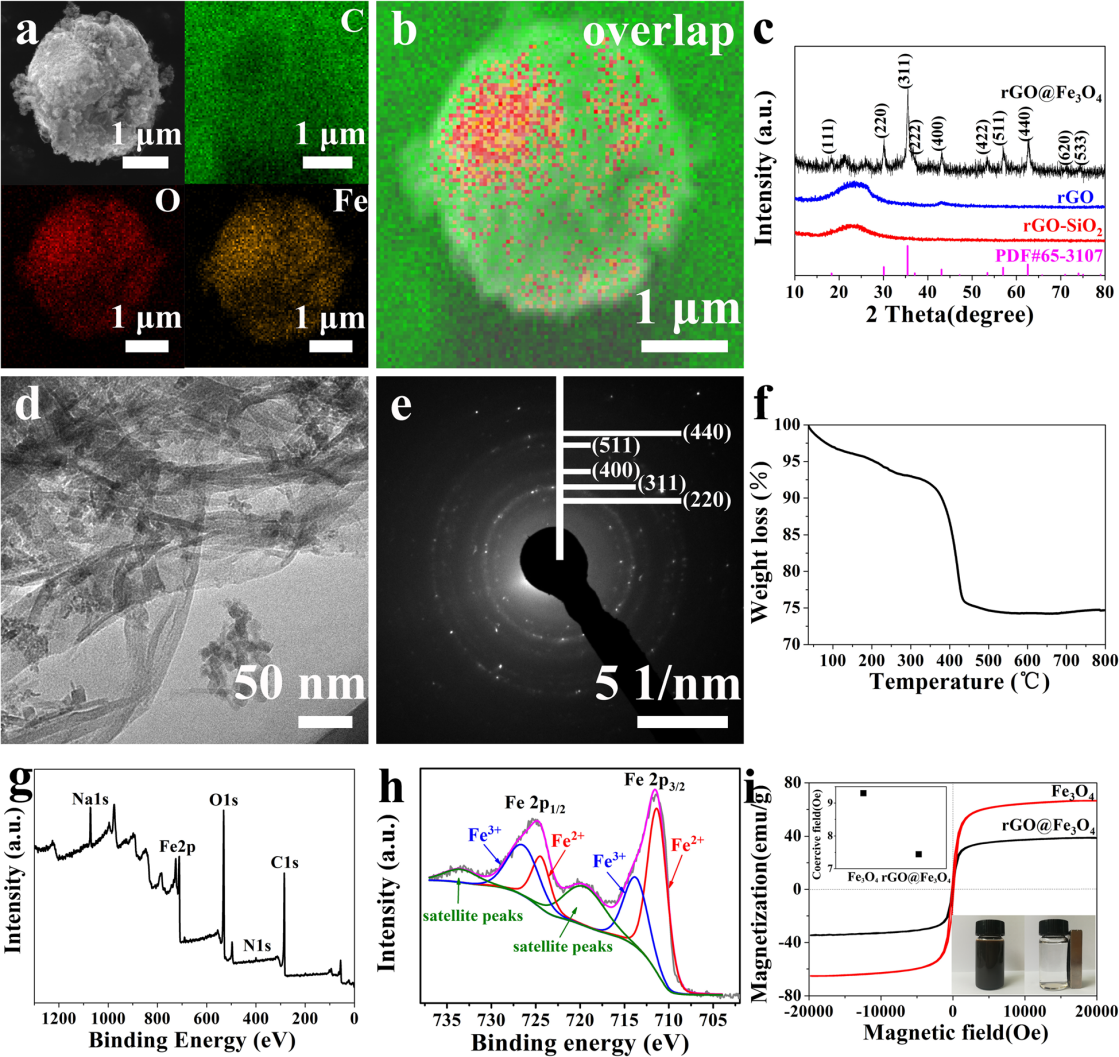
Structure and composition characterization of rGO@Fe3O4. (**a**, **b**) SEM with EDS mapping pictures of rGO@Fe_3_O_4_ microspheres: C, Fe, and O elements; (**c**) XRD patterns of rGO-SiO_2_, rGO and rGO@Fe_3_O_4_ microspheres; (**d**, **e**) SEAD images of rGO@Fe_3_O_4_ microspheres; (**f**) TG curves of rGO@Fe_3_O_4_ microspheres; (**g**, **h**) XPS spectra of rGO@Fe_3_O_4_ microspheres; (**i**) Magnetic hysteresis loops of the Fe_3_O_4_ and rGO@Fe_3_O_4_ microspheres (inset of top shows coercive field values (Hc) of samples, and inset of the bottom shows their suspensions before and after magnetic separation by an external magnet)

To further confirm the successful preparation of rGO@Fe_3_O_4_, SEM with EDS was employed to investigate the structure and composition of the microspheres. The EDS images of rGO@Fe_3_O_4_ were characterized by visualizing the inelastically scattered electrons in the energy loss windows for elemental O, Fe, and C, and the different color areas represent O, Fe, and C enriched locations in real structures, respectively. As shown in Fig. 2a and b, Fe and O were widely distributed in rGO@Fe_3_O_4_ microspheres with high loading density. Figure 2d confirmed that the Fe_3_O_4_ nanoparticles uniformly dispersed in the rGO with a diameter of about 18 nm, resulting in a sharp decrease of pore size in rGO@Fe_3_O_4_ microspheres. The selected area electron diffraction (SAED) pattern further verified the presence of Fe_3_O_4_ in rGO (Fig. 2e), the characteristic resonance in 2.98 nm, 2.53 nm, 2.09 nm, 1.62 nm, and 1.49 nm face spacing assigned to the 220, 311, 400, 511, and 440 planes of the face-centered-cubic phase of Fe_3_O_4_, respectively. The peaks appeared at 220, 311, 400, 511, and 440 corresponding to Fe_3_O_4_ were also detected in the XRD spectra, which was consistent with SAED results (Fig. 2c). However, it is reported that Fe_3_O_4_ and γ-Fe_2_O_3_ could not be distinguished by the XRD pattern independently for the same location of characteristic peaks [35]. XPS result showed that the predominant peaks at 725.9/724.5 eV and 714.1/711.0 eV, corresponding to Fe2p_1/2_ and Fe 2p_3/2_ of the rGO@Fe_3_O_4_ (Fig. 2g, h), respectively, indicating the coexistence of Fe^3+^ and Fe^2+^ in Fe_3_O_4_ [36]. Thermogravimetric (TGA) analysis was performed to monitor the thermal degradation behavior of rGO in rGO@Fe_3_O_4_ microspheres by heating the sample to 800 °C and cooling down to 100 °C in an air atmosphere (Fig. 2f). The mass loss curve showed two distinct mass loss regions including the dehydration region (40-300 °C) and the devolatilization region (300-800 °C) of rGO in rGO@Fe_3_O_4_, carbon content calculated from the sample was 25.6 wt.%.

The magnetic properties of rGO@Fe_3_O_4_ microspheres were investigated using a superconducting quantum interference device. The magnetic field was conducted with a scanning range from −20,000 to 20,000 Oe at room temperature. Figure 2i shows the saturation magnetization (Ms) value and coercive field (Hc) value of Fe_3_O_4_ are 66.6 emu g^−1^ and 9.3 Oe. After loading Fe_3_O_4_ onto rGO, the Ms value and Hc value of the rGO@Fe_3_O_4_ microspheres decreased to 33.9 emu g^−1^ and 7.44 Oe. The remarkably decrease in magnetic saturation can be contributed to the diamagnetic properties of rGO in rGO@Fe_3_O_4_ microspheres. Moreover, The selective agglomeration ability of rGO@Fe_3_O_4_ microspheres was performed intuitively by magnetic separation experiment. The suspensions of the Fe_3_O_4_ and rGO@Fe_3_O_4_ microspheres were put into the vial with an external magnet for 2 min, the suspensions can be concentrated to the magnet side and the aqueous solution became transparent. When the magnet was taken away, the rGO@Fe_3_O_4_ microspheres were dispersed uniformly again after slowly shaking, indicating that rGO@Fe_3_O_4_ microspheres holding the merit of good water-dispersive ability. The excellent water-dispersive ability and magnetic response property payed the way for the magnetic targeted application of rGO@Fe_3_O_4_ as drug carries in cancer treatment.

### Photothermal Effect Analysis

**Fig. 3:**
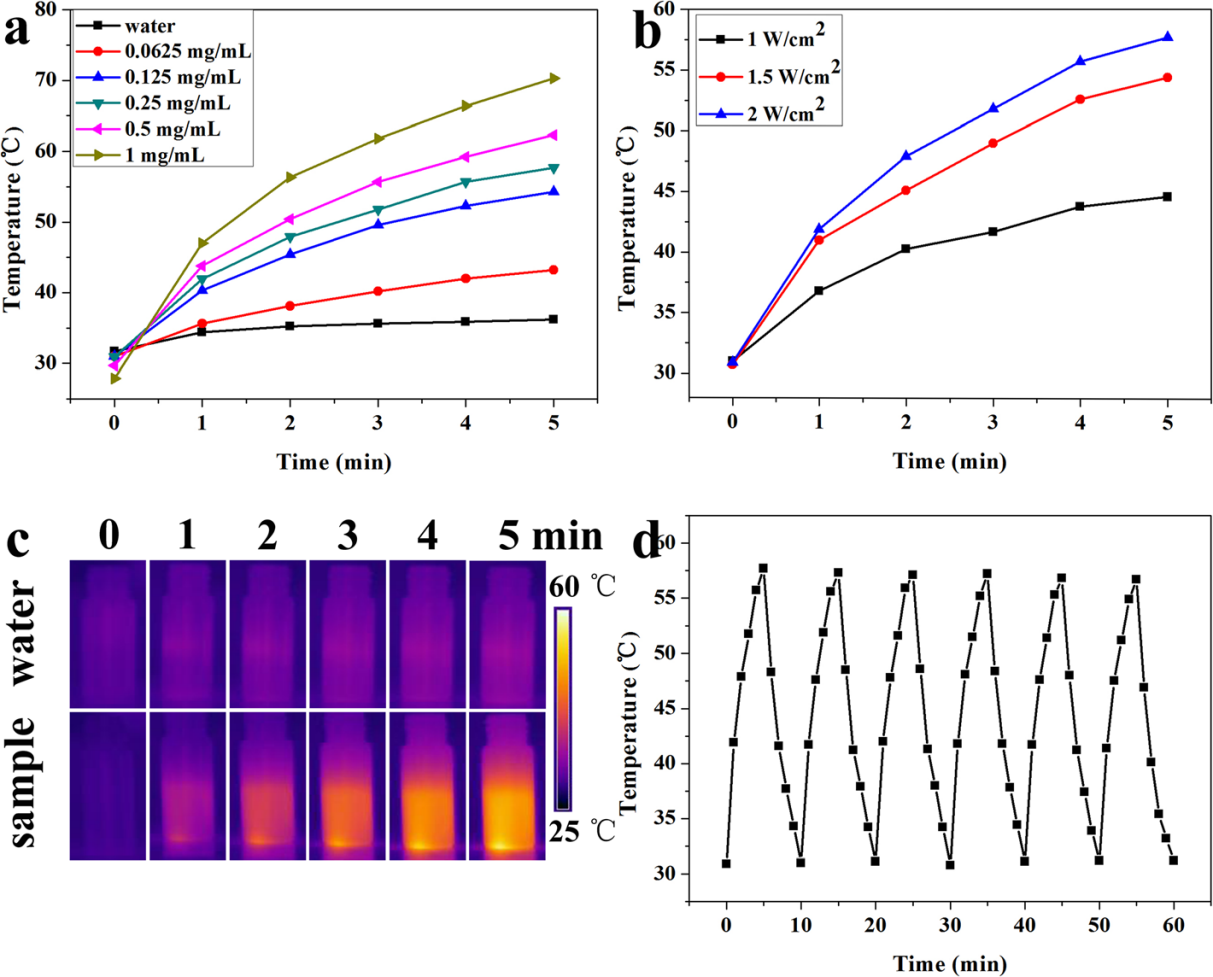
Photothermal effects of rGO@Fe3O4. **a** Concentration-dependent temperature change of rGO@Fe_3_O_4_ solutions at different concentrations (0.0625, 0.125, 0.25, 0.5, and 1 mg mL^−1^) under 808 nm irradiation at 2 W cm^−2^ for 5 min. **b** Power-dependent temperature response of 0.25 mg mL^−1^ rGO@Fe_3_O_4_ solution under the irradiation of an 808 nm NIR laser for 5 min (1 W cm^−2^, 1.5 W cm^−2^, 2 W cm^−2^). **c** Infrared thermal images of rGO@Fe_3_O_4_ solution at 0, 1, 2, 3, 4, and 5 min intervals stimulated at 808 nm (2 W cm^−2^). **d** Temperature increases of rGO@Fe_3_O_4_ (0.25 mg mL^−1^) solution during 6 successive cycles of laser on/off under 808 nm irradiation at 2 W cm^−2^

**Fig. 4:**
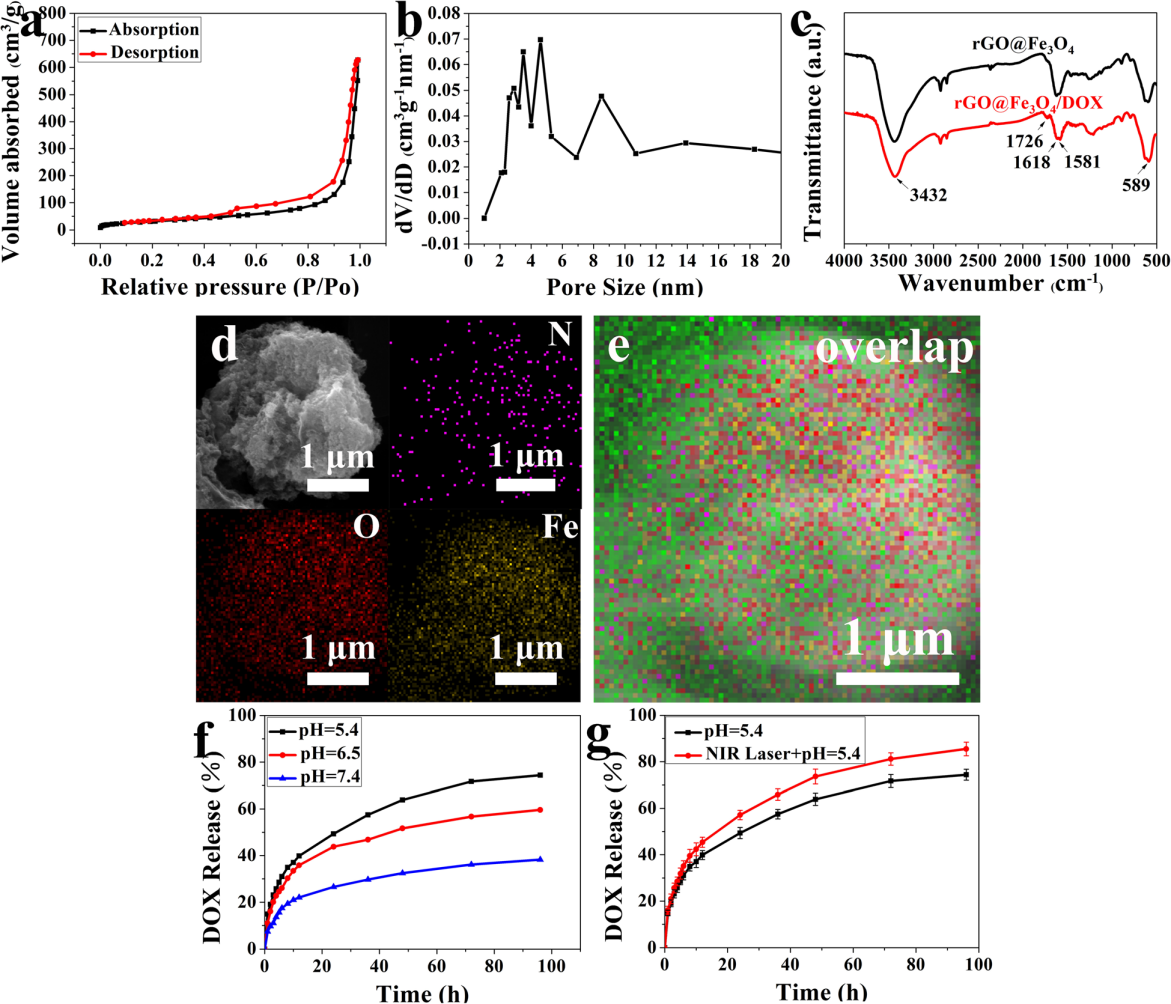
The surface area and pore-size of rGO@Fe3O4 microspheres, DOX loading and release behaviors. **a** Nitrogen adsorption-desorption isotherms of rGO@Fe_3_O_4_. **b** Pore-size distribution of rGO@Fe_3_O_4_. **c** FTIR spectra of rGO@Fe_3_O_4_ and rGO@Fe_3_O_4_/DOX. **d**, **e** SEM and mapping images of N, Fe, and O of rGO@Fe_3_O_4_/DOX microspheres. **f** Drug-release kinetic curves obtained at different pH values of rGO@Fe_3_O_4_ microspheres. **g** NIR-responsive DOX release kinetic curves

Considering the deeper penetration into the tissue and less damage to surrounding tissues of NIR, NIR-responsive photothermal therapy was often employed to tumor treatment. Hence, the photothermal transformation behavior of rGO@Fe_3_O_4_ aqueous solutions at different concentrations and different power densities were recorded under NIR laser irradiation at 808 nm for 5 min. Figure 3a, b showed that the temperature increase of rGO@Fe_3_O_4_ was highly dependent on the concentration and the laser power density. When the concentration of the microspheres was up to 1 mg mL^−1^, the temperature raised up from 27.9 to 70.3 °C under NIR laser irradiation for 5 min at 2 W cm^−2^, while the temperature for PBS group just raised up from 31.7 to 36.2 °C. The high photothermal conversion efficiency of rGO@Fe_3_O_4_ will have a great potential for tumor photothermal therapy according to previous report that protein degeneration and DNA damage in cell will happen (happened) upon exposure to 50 °C for 4 to 6 min [21, 37]. To intuitively display the photothermal transformation behavior of rGO@Fe_3_O_4_, IR thermography was performed and the results were shown in Fig. 4c. The rGO@Fe_3_O_4_ solution with the concentration of 1 mg mL^−1^ was quickly increased to 70.3 °C after NIR irradiation for 5 min, while the water group has no obvious changes, which was consistent with the thermometry results. Furthermore, the photothermal stability of the rGO@Fe_3_O_4_ was studied by performing laser on/off procedure with an 808 nm laser at 2 W cm^−2^ for six cycles (Fig. 3d). The identical temperature increasement was obtained, indicating the perfect NIR photothermal stability of rGO@Fe_3_O_4_ composites. These results demonstrated that rGO@Fe_3_O_4_ microspheres holding great promise as a photothermal agent for photothermal therapy in cancer.

### Drug Loading and Release

The surface area and pore-size of rGO@Fe_3_O_4_ were evaluated by BET and BJH analyses (Fig. 2a, b). N_2_ adsorption-desorption curve type was isothermal IV type, and the surface area and pore size were 120.7 m^2^ g^−1^, 2-8 nm and 1.012 cm^3^ g^−1^, respectively. The results showed that rGO@Fe_3_O_4_ possessed mesoporous channels and average pore size distribution, exhibiting great potential for anti-tumor drug loading. Then, the rGO@Fe_3_O_4_ microspheres with porous structure were served to load a model chemotherapeutic drug doxorubicin by simply mixing and slight sonication. The ATR-FTIR analysis further verified the stable incorporation of DOX in rGO@Fe_3_O_4_ due to the characteristic resonance of -COOH and benzene groups of DOX at 1726 cm^−1^ and 1618 cm^−1^ (Fig. 4c). Scanning electron microscopy (SEM) observation showed that the new signals of N elements assigned to DOX distributed uniformly in microsphere after DOX loading (Fig. 4d, e). Moreover, the DOX loading efficiency (LE) and loading capacity (LC) of rGO@Fe_3_O_4_/DOX were 92.15% and 18.43%, respectively. The remarkably higher LCs of rGO@Fe_3_O_4_/DOX than many drug-carriers can be contributed to extremely high surface areas and pore sizes [19]. The high LE of rGO@Fe_3_O_4_/DOX may be attributed to two aspects, one is that rGO@Fe_3_O_4_ can interact with DOX by strong π–π stacking between sp2-hybridized π bonds of rGO@Fe_3_O_4_ and the quinine portion of DOX [21], and another one may be that they can form hydrogen bonding between the carboxylic acid (–COOH), hydroxyl (–OH) groups of rGO@Fe_3_O_4_ and the amine (–NH_2_), hydroxyl (–OH) groups of DOX. Then, we monitored DOX release behavior in PBS at pH 7.4, 6.5, and 5.4, to mimic the extracellular environments of tumor and normal tissues. As indicated in Fig. 4f, the release rate of DOX was accelerated when the pH was adjusted from 7.4 to 5.4, and the sustained DOX release at pH 5.4 can be up to 73% after 98 h treatment. Therefore, the cumulative release profile of DOX from rGO@Fe_3_O_4_ exhibited a pH-dependent manner. This accelerated release under acidic conditions could be due to the partial protonation of the hydroxyl and amine groups of DOX, leading to higher drug solubility and weakening of hydrogen bonds between DOX and graphene [38]. Furthermore, we also studied the NIR-responsive DOX release behavior in vitro. As indicated in Fig. 4g, DOX release was accelerated by NIR-radiation and the release rate of DOX was up to 85%. This pH and NIR stimuli-responsive behavior plays an important role in effective drug delivery towards the tumor site.

### In Vitro Cell Uptake

**Fig. 5:**
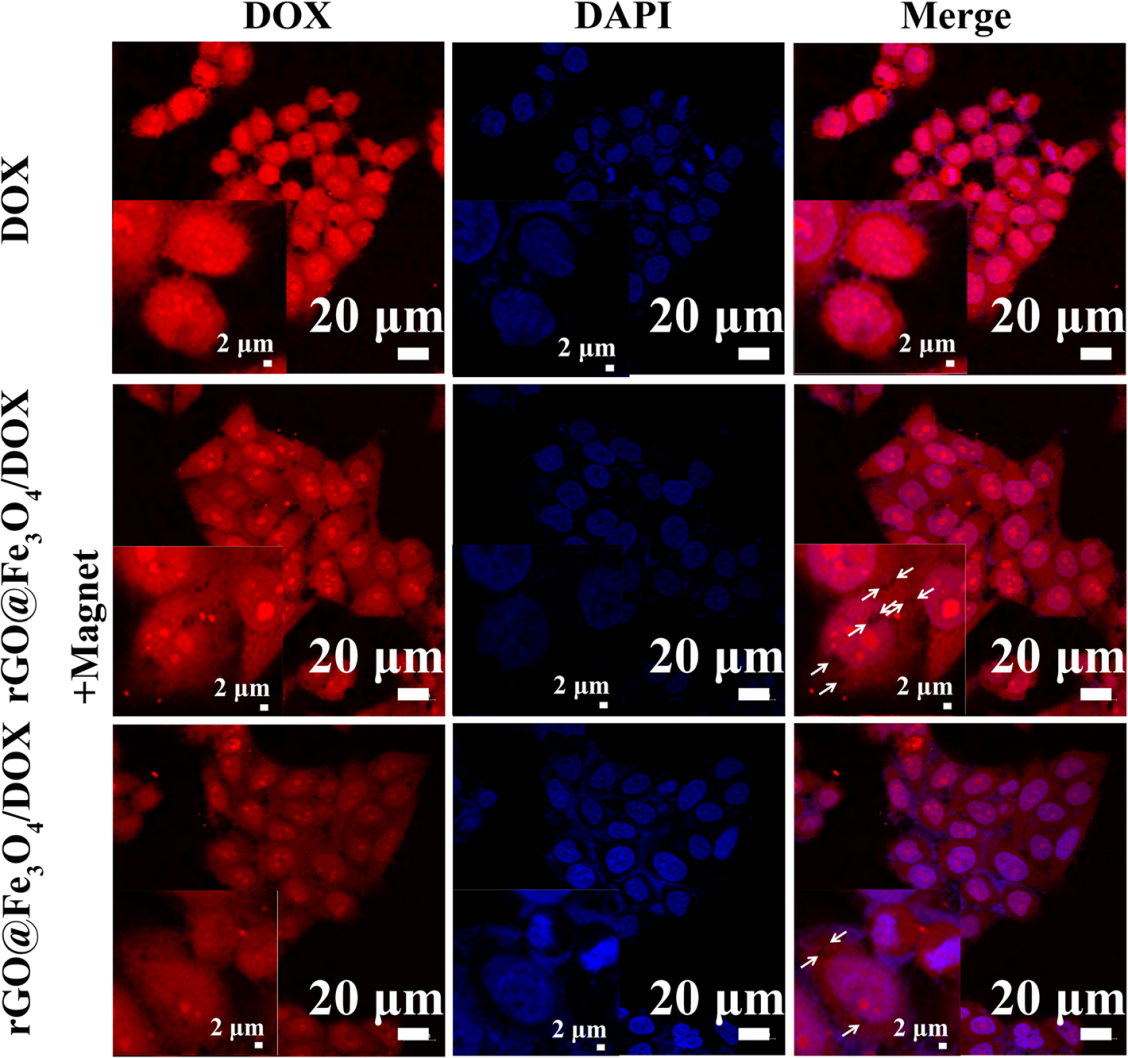
Magnetic target evaluation of rGO@Fe3O4-DOX microspheres. CLSM images of rGO@Fe_3_O_4_/DOX incubated HeLa cells with and without magnet (insets shows the image at high magnification)

To verify the magnetic targeting ability of Fe_3_O_4_ in rGO@Fe_3_O_4_ microsphere, the cellular uptake experiments with or without magnetic field treatment was qualitatively investigated by confocal laser scanning microscopy (CLSM). Hela cells were incubated with rGO@Fe_3_O_4_/DOX for 4 h and the nuclei of Hela were stained by DAPI. The results in Fig. 5 showed that the black spot corresponding to rGO@Fe_3_O_4_ microsphere and obvious intracellular red fluorescence signals assigned to DOX were observed in the rGO@Fe_3_O_4_ group with a magnetic field treatment. In contrast, there was less black spot and weaker DOX fluorescence can be found when rGO@Fe_3_O_4_ group without magnetic field loading. The explanation may be that the black spot attributed to rGO@Fe_3_O4 internalized into the cell could be promoted by magnet. The results indicate that Fe3O4 in rGO@Fe_3_O4/DOX could specifically target Hela cells efficiently and significantly enhance the cell internalization of microspheres, demonstrating favorable magnetic targeting ability of the drug delivery system in cancer therapy.

### In Vitro Cytotoxicity Analyses

**Fig. 6:**
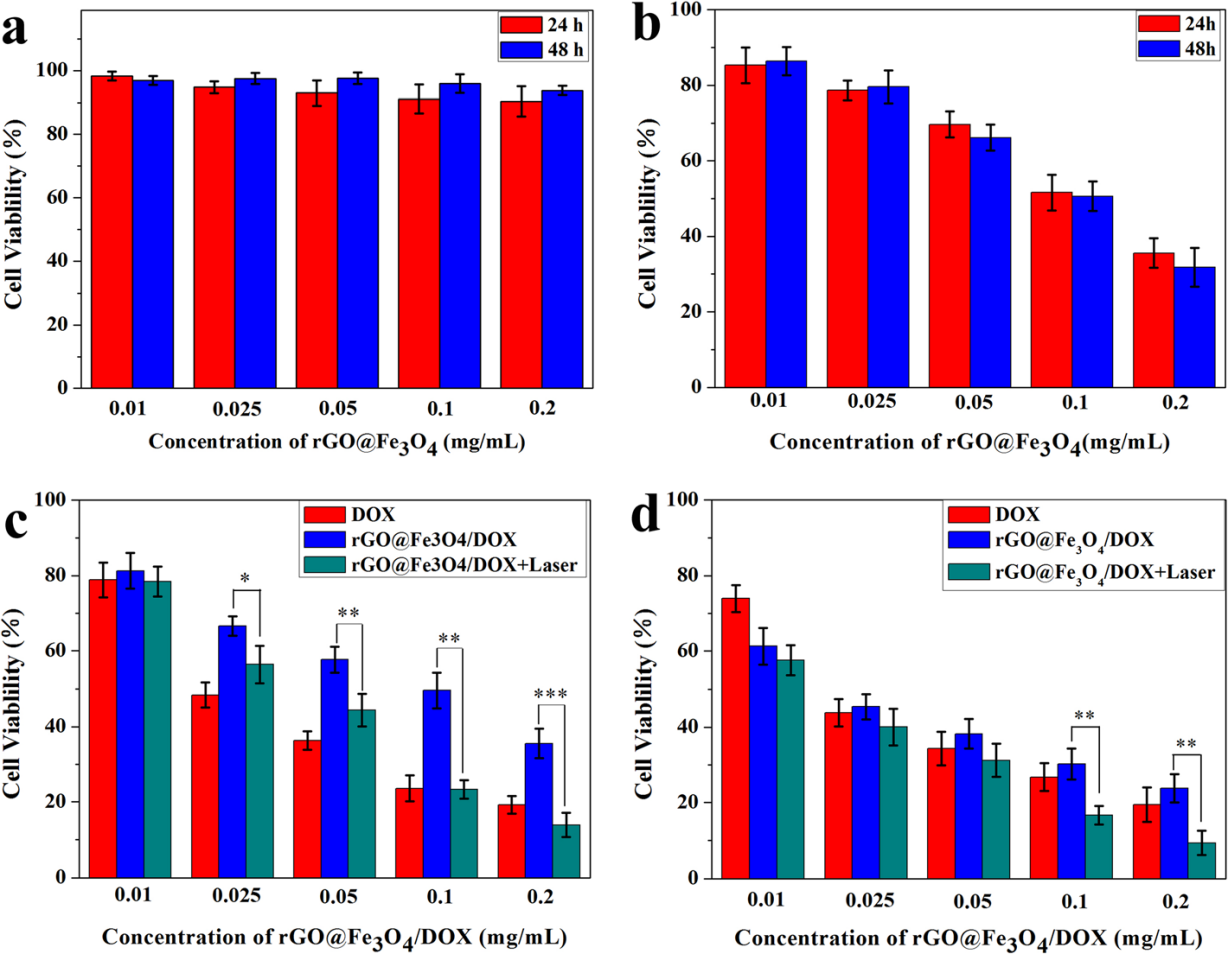
The biocompatibility and the therapeutic efficacy of single photothermal therapy or combined photothermal-chemotherapy. **a** Cell viability of Hela cells cultured with rGO@Fe_3_O_4_ for 24 h and 48 h. **b** Cell viability of Hela cells cultured with or without NIR irradiation at different concentrations of rGO@Fe_3_O_4_ for 24 h and 48 h. (**c**, **d**) Cell viability of Hela cells cultured with free DOX, rGO@Fe_3_O_4_/DOX microspheres for 24 h and 48 h with and without NIR irradiation (808 nm, 2 W cm^−2^) (*p < 0.05, **p < 0.01, ***p < 0.001)

The biocompatibility of rGO@Fe_3_O_4_ was evaluated using CCK-8 assay towards Hela cells. As shown in Fig. 6a, after incubation with rGO@Fe_3_O_4_ at a wide range of different concentrations, the cell viability was also higher than 90% even at high concentrations up to 200 μg mL^−1^, the results indicated that rGO@Fe_3_O_4_ exhibits a high biocompatibility and could be served as an efficient drug delivery platform. The photothermal therapy efficacy of rGO@Fe_3_O_4_ was further investigated after incubation with Hela cells for 24 h and 48 h under NIR light irradiation (808 nm NIR laser, 10 min). As shown in Fig. 6b, the phototoxicity was clearly dose-dependent upon NIR stimulation, and cell viability decreased from 90.37 to 35.52% at 24 h, and from 93.77 to 31.75% at 48 h, implying that rGO@Fe_3_O_4_ had excellent phototoxicity and hold great promise in photothermal therapy. To estimate the synergic therapeutic efficacy of photothermal-chemotherapy, the cytotoxicity of rGO@Fe_3_O_4_/DOX towards Hela cells with and without NIR irradiation were studied. As shown in Fig. 6c, d, the cell viability showed concentration-dependent and time-controlled manner. Approximately 65% and 80% of Hela cells were killed by rGO@Fe_3_O_4_/DOX without NIR irradiation and DOX at 24 h, the decreased tumor-killing ability of rGO@Fe_3_O_4_/DOX compared to free DOX may be due to the delayed DOX release behavior of rGO@Fe_3_O_4_/DOX microspheres. After NIR laser irradiation (808 nm NIR laser, 10 min), rGO@Fe_3_O_4_/DOX with laser group killed more than 86% cells at an equivalent dose of DOX (30 μg mL^−1^). Similar results could be observed after the same treatment cells for 48 h, the decrease in the cell viability of DOX, rGO@Fe_3_O_4_/DOX, rGO@Fe_3_O_4_/DOX with NIR irradiation group was 80%, 76%, and 90%, respectively, indicating a synergistic effect of the combined photothermal therapy and chemotherapy.

## Conclusions

In summary, we explored a facile strategy to construct rGO-based drug delivery platform rGO@Fe_3_O_4_/DOX for synergistic photothermal-chemotherapy. rGO@Fe_3_O_4_/DOX microsphere exhibited excellent NIR-triggered PTT effect and perfect NIR photothermal stability. The Fe_3_O_4_ on the microspheres ensured excellent tumor cells targeting ability. DOX could be encapsulated into rGO@Fe_3_O_4_ with an ultrahigh drug-loading capacity and a pH-responsive drug release behavior could be simultaneously achieved. In addition, an enhanced antitumor efficiency was achieved when a combination of chemotherapy and photothermal therapy. Therefore, this multifunctional drug delivery platform could be a promising candidate for tumor targeting and combinatorial cancer therapy in the future.

## Data Availability

The data and the analysis in the current work are available from the corresponding authors on reasonable request.

## Abbreviations

DDS: Drug delivery system
NIR: Near-infrared
GO: Graphene oxide
DOX: Doxorubicin
DMEM: Dulbecco’s minimum essential medium
DAPI: 4',6-diamidino-2-phenylindole
CCK-8: Cell counting kit-8
SEM: Scanning electron microscopy
TEM: Transmission electron microscope
XRD: X-ray diffraction system
XPS: X-ray photoelectron spectroscopy
FTIR: Fourier transform infrared spectroscopy
TGA: Thermogravimetric analyzer
LE: Loading efficiency
LC: Loading capacity
